# Comparison of Chamfer and Deep Chamfer Preparation Designs on the Fracture Resistance of Zirconia Core Restorations

**DOI:** 10.5681/joddd.2011.009

**Published:** 2011-06-14

**Authors:** Ezatollah Jalalian, Roghayeh Rostami, Berivan Atashkar

**Affiliations:** ^1^ Associate Professor, Department of Prosthodontics, Dental School, Islamic Azad University, Tehran, Iran; ^2^ Dentist, Private Practice, Tehran, Iran

**Keywords:** CAD/CAM, dental restoration, fracture strength, zirconium oxide

## Abstract

**Background and aims:**

One of the major problems of all-ceramic restorations is their probable fracture under occlusal force. The aim of the present in vitro study was to compare the effect of two marginal designs (chamfer and deep chamfer) on the fracture resistance of all-ceramic restorations, CERCON.

**Materials and methods:**

This in vitro study was carried out with single-blind experimental technique. One stainless steel die with 50’ chamfer finish line design (0.8 mm deep) was prepared using a milling machine. Ten epoxy resin dies were prepared. The same die was retrieved and 50' chamfer was converted into a deep chamfer design (1 mm). Again ten epoxy resin dies were prepared from the deep chamfer die. Zirconia cores with 0.4 mm thickness and 35 µm cement space were fabricated on the epoxy resin dies (10 chamfer and 10 deep chamfer samples). The zirconia cores were cemented on the epoxy resin dies and underwent a fracture test with a universal testing machine and the samples were investigated from the point of view of the origin of the failure.

**Results:**

The mean values of fracture resistance for deep chamfer and chamfer samples were 1426.10±182.60 and 991.75±112.00 N, respectively. Student’s t-test revealed statistically significant differences between the groups.

**Conclusion:**

The results indicated a relationship between the marginal design of zirconia cores and their fracture re-sistance. A deep chamfer margin improved the biomechanical performance of posterior single zirconia crown restorations, which might be attributed to greater thickness and rounded internal angles in deep chamfer margins.

## Introduction

One of the major problems of all-ceramic restorations is their probable fracture under occlusal and lateral forces.^1^ The majority of restorations contain metal which brings about toxic, chemical and allergic affects. The difference between their color and that of the natural tooth is another problem. Most people prefer tooth-colored crowns. All-ceramic crowns have esthetic and biocompatibility.^2^ In recent years such restorations have been used in posterior restorations. However, some crown fractures due to the relatively low mechanical resistance of ceramic crowns have been reported, which might be attributed to the magnitude of biting forces applied on premolars and molars and to the inherent brittleness of ceramics.^3,4^Ceramic materials are particularly susceptible to tensile stresses, and mechanical resistance is also strongly influenced by the presence of superficial flaws and internal voids. Such defects might represent the sites of crack initiation. This phenomenon may be influenced by different factors, such as marginal design and thickness of the restoration, residual processing stress, magnitude and direction and frequency of the applied load, elastic modulus of restoration components, restoration?cement interfacial defects, and oral environmental effects.^5^In one research, finite element analysis (FEA) was used to study stress distribution during mastication in maxillary second premolars restored with metal-ceramic crowns and compare them to non-restored teeth. High stresses were recorded at the cervical line of restored teeth within the dentin-metal interface and within the ceramic-metal interface.^6^ The FEA method was used to study stress distribution in the lower first molars restored with all-ceramic crowns. The results of that study revealed concentration of stress at the cervical area.^7^ The aim of the present study was to evaluate the effect of marginal design of crowns on improved mechanical performance of CERCON crowns from a clinical point of view. Such a condition can be achieved preparing a deep chamfer margin in crowns instead of a chamfer and shoulder margin. Florian Beuer^8^ suggested that shoulder margin has a greater fracture resistance than deep chamfer and chamfer margin. Sadan et al^9^ proposed that both these types of finish lines are considered to be adequate for the tooth. However, Di Lorio et al^10^ suggested that the shoulder margin could improve the biomechanical performance of single-crown alumina restorations. De Jager et al^11^ discovered that for long-lasting restorations in posterior region it is advisable to make a chamfer with collar preparation. Cho L et al^12^ found out that the fracture strength of chamfer finish line (0.9, 1.2 mm) was greater than 1.2 mm rounded end shoulder and 1.2 shoulder finish line. Potiket et al^13^ suggested that a 1-mm deep shoulder finish line with a rounded internal line angle has good fracture strength for the natural teeth restored with all-ceramic crowns. Rammersberg et al^14^ discovered that a minimally invasive 0.5-mm axial chamfer tooth preparation has the greatest stability for posterior metal-free crowns. The aim of the present in vitro study was to compare the fracture resistance under a cyclic load applied to chamfer and deep chamfer margins of zirconia crowns.

## Materials and Methods

This in vitro single-blind experimental study was carried out using 1 machined standard stainless steel die with a height of 7 mm and a diameter of 5 mm.^15^The marginal area of the die was prepared with 50' chamfer finish line (0.8 mm deep).^16,17^ The axial walls were 10° convergent (Figure 1).^15^ Impressions were poured in Epoxy resin CW2215 (Hunstman-Germany). Afterwards, the standard die was converted into a deep chamfer with a depth of 1 mm(Figures 2a,b).^16,17^ Again 10 polyvinylsiloxane impressions were made and ten epoxy resin dies were created from these impressions (Figure 2c,d).^8,10^ Twenty copings were produced of a partially sintered ZrO_2_ ceramic material using CAD/CAM technology (Cercon Smart Ceramics, DeguDent, Hanau, Brain, DeguDent). The copings with 0.4 mm thicknesses^8^and 35 µm of cement space^8^weremilled out from the pre-sintered ZrO_2_ and the Cercon (DeguDent) heat-sintered them at 1350°C for 6 hours. Since the coping mainly determinates the overall resistance to fracture of veneered crown^5,18^porcelain veneering was omitted. The copings were evaluated visually; those with margin deemed visually unacceptable were rejected and another coping was made instead. Each coping was then cemented on its definitive die with GI (GC Gold Labled, Tokyo, Japan).^14^ Finger pressure was applied during the setting time.^24^ After cementation, excess luting agent was removed and the samples were stored in a saline solution at room temperature for 24 hours. Mechanical tests were carried out using a universal testing machine (GOTECH AI-700LAC, Arsona, USA). The load was applied at the center of the occlusal surface along the long axis with a crosshead speed of 0.5 mm/min until fracture occurred.^19^ The fracture load data were automatically recorded using Vista software. The samples were investigated from the point of view of the origin of the failure (Figure 2e,f). Data was analyzed with student's t-test at a significance level of P<0.05.

 Figure 1. Diagram of chamfer (a) and deep chamfer (b) preparations.

**a:**
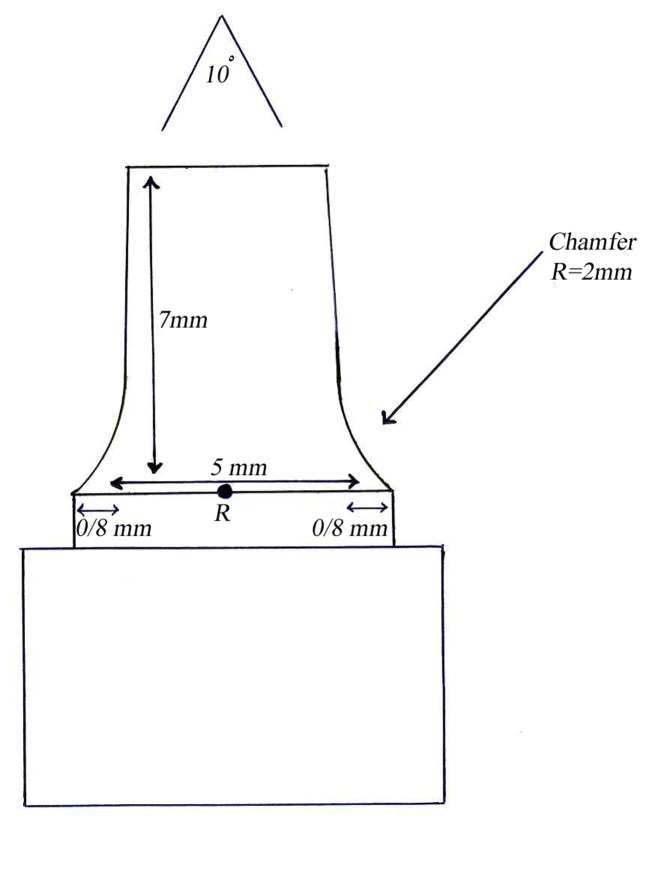

**b:**
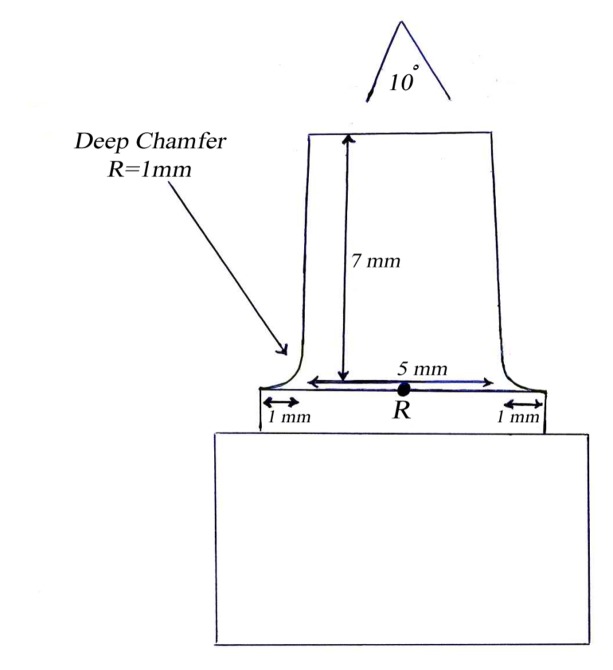

 Figure 2. Standard dies of chamfer (a) and deep chamfer (b) preparations. Epoxy resin dies with chamfer (c) and deep chamfer (d) margins. Fracture area in chamfer (e) and deep chamfer (f) margins.

**a:**
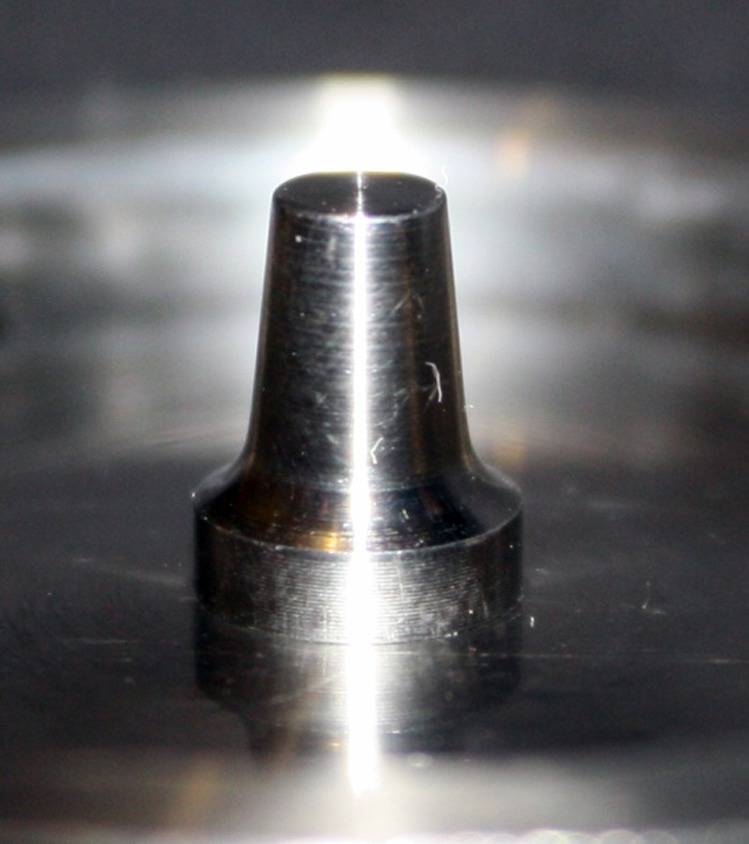

**b:**
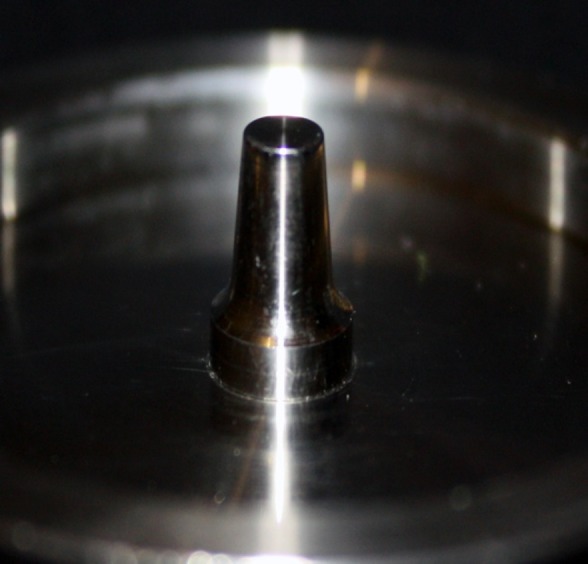

**c:**
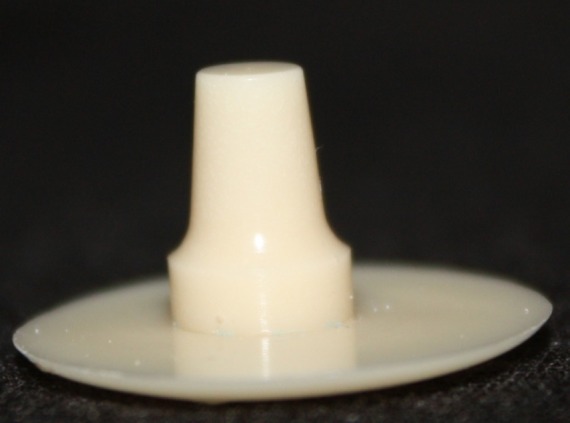

**d:**
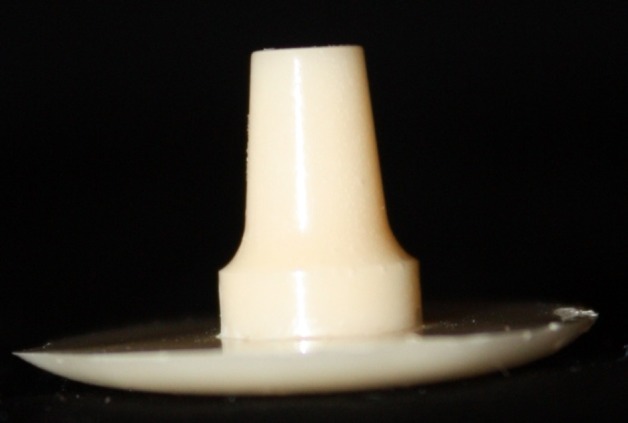

**e:**
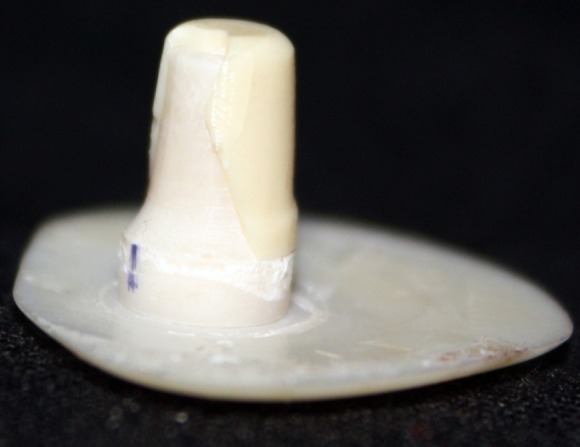

**f:**
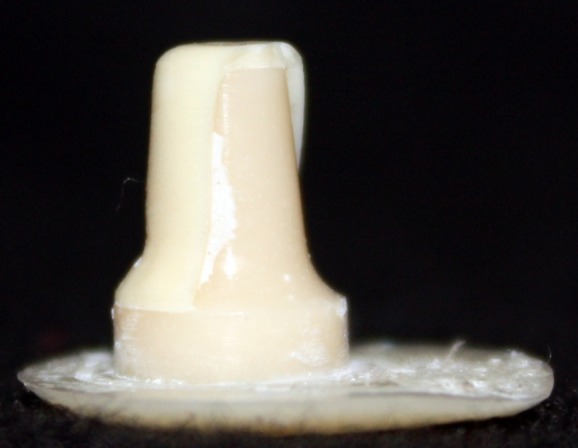

## Results

The mean ± SD of fracture resistance were 1426.10 ± 182.60 and 991.75 ± 112.00 N for the deep chamfer and chamfer margins, respectively. Not only the maximum but also the minimum fracture resistances of two groups were more than intra-oral loads. Student's t-test revealed statistically significant differences between the groups (P=0.05) (Table 1). This study was carried out with 95% confidence interval; Kaplan–Meir graph showed that deep chamfer margin tolerates more cracks till fracture than chamfer margin (Figure 3), which might be attributed to greater thickness in deep chamfer margins.

**Table 1:** Fracture resistance of chamfer and deep chamfer edge zirconia cores

Margin design	N	Mean	Std. Deviation	Std. Error	95% Confidence Interval for Mean		Minimum	Maximum
					Lower Bound	Upper Bound		
Deep chamfer	10	1426.100	182.60671	57.74531	1295.4710	1556.7290	1100.00	1656.00
Chamfer	10	991.7500	112.00088	25.04416	939.3320	1044.1680	813.00	1196.00

 Figure 3. Error bar and Kaplan–Meir graph for fracture resistance of deep chamfer and chamfer preparations.

**finish line:**
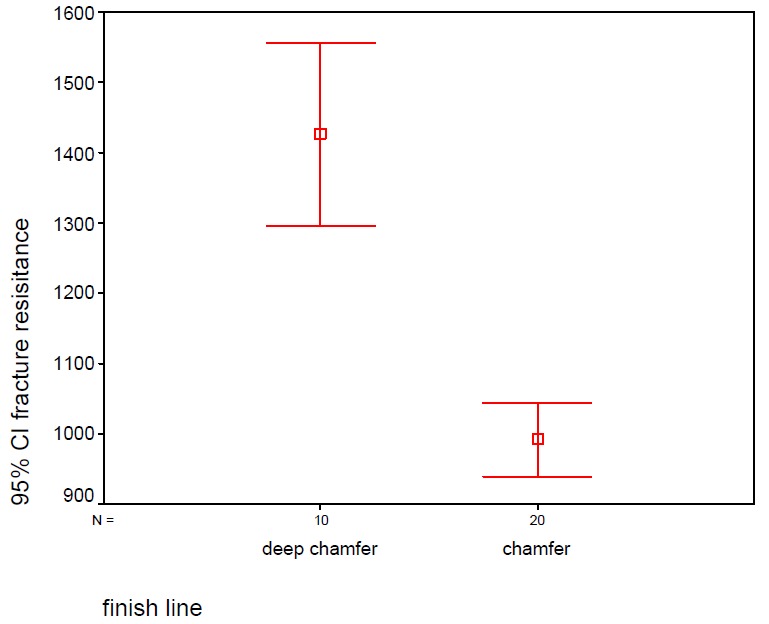

**RESIST:**
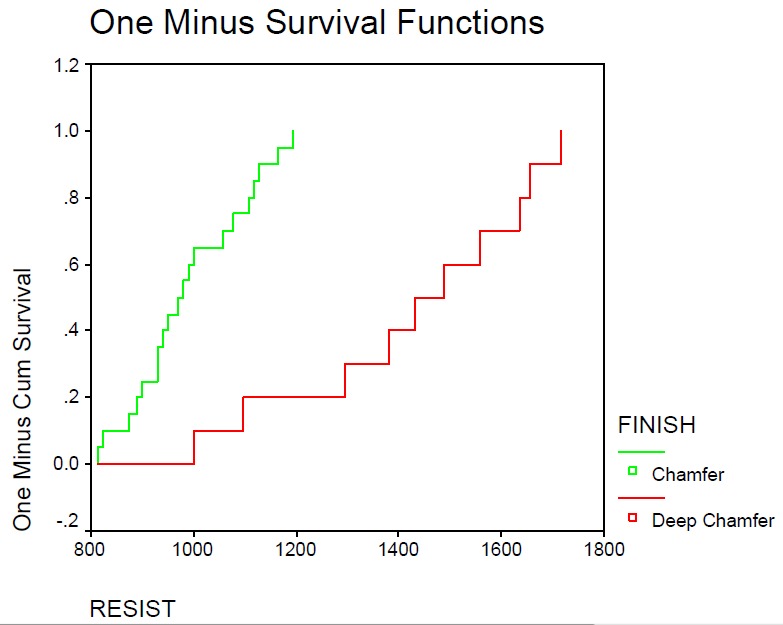

## Discussion

One of the major problems of all-ceramic restorations is their probable fracture under occlusal and lateral forces.^1^The majority of restorations contain metal which brings about biologic problems and have no esthetic appearance.^2^ This study compared fracture resistance of chamfer and deep chamfer margins of CERCON crowns under a cyclic load. Student's t-test revealed statistically significant differences between the two groups; fracture resistance of deep chamfer margin was more than that of chamfer margin. Elastic modulus of the supported materials of the core affected the fracture resistance of the core.^20^ Therefore, in this study, we used epoxy resin dies that are much better brass dies.^21^ Another difference from clinical conditions is the unknown nature of the bond between the luting agent and die material. It is reasonable to suppose that the presence of a hybrid layer at the dentin-cement interfaces influences the biomechanical behavior of the core/supporting die system. However, both of these factors equally influenced the samples in the present study. Therefore, it is possible to make a comparison between the two groups. Fracture resistance of the two groups are more than the occlusal forces so we could use all of these marginal designs successfully in the posterior all-ceramic crowns, which are very good substitutes for PFM crowns. However, there was a statistically significant difference between the two groups, revealing that the deep chamfer has more fracture resistance than chamfer margin, which might be attributed to greater thickness in deep chamfer margins that can bear load better than chamfer margins. We used resin cements for cementation, so we had a strong unity in the margins that provided strength against fracture.^22^ It seems deep chamfer can bear load better, making it more fracture resistant than chamfer margin.

## Conclusion

Both marginal designs had high fracture resistance that is more than biting forces so we could use both; however, because of higher fracture resistance of deep chamfer margins, this finish line is recommended to improve the biomechanical performance of posterior single zirconia restorations.
